# Combined therapy with EGFR TKI and gambogic acid for overcoming resistance in *EGFR*-T790M mutant lung cancer

**DOI:** 10.3892/ol.2015.3599

**Published:** 2015-08-12

**Authors:** CHENGDE WANG, WEI WANG, CHAOYANG WANG, YIJUN TANG, HUI TIAN

**Affiliations:** 1 Department of Thoracic Surgery, Qi Lu Hospital, Shandong University, Jinan, Shandong 250012, P.R. China; 2 Department of Thoracic Surgery, Yantai Yuhuangding Hospital, Yantai, Shandong 264000, P.R. China

**Keywords:** gefitinib, gambogic acid, tyrosine kinase inhibitor, non-small cell lung cancer

## Abstract

Although patients with non-small cell lung cancer (NSCLC) experience an initial response to the epidermal growth factor receptor (EGFR) tyrosine kinase inhibitor gefitinib, those individuals with activating mutations in EGFR develop resistance. Gambogic acid (GA), a polyprenylated xanthone, has strong antitumor activities. In the present study, the therapeutic efficacy of gefitinib with GA was evaluated in a gefitinib-resistant NSCLC model. The NCI-H1975 cell line with EGFR-T790M mutation was subcutaneously injected into immunocompromised mice. The mice were randomly assigned to receive treatment with gefitinib, GA, gefitinib plus GA, or vehicle for 4 weeks, then all mice were sacrificed and their tumor tissues were subjected to caspase activity detection and western blot analysis. Gefitinib and GA alone slightly inhibited the tumor growth of NCI-H1975. However, the combined treatment significantly enhanced their antitumor effects, without any marked adverse events. In addition, gefitinib plus GA enhanced the level of apoptosis in the tumor tissues. Western blot analysis also revealed that the combination treatment reduced the phosphorylation level of AKT, MEK1/2 and ERK1/2, while an increased expression ratio of Bax/Bcl-2 was observed. In the current study, gefitinib in combination with GA resulted in antitumor growth in the EGFR-T790M secondary mutation NCI-H1975 tumor model due to an enhanced apoptotic effect. This novel therapeutic strategy may be a practical approach for the treatment of patients who show gefitinib resistance.

## Introduction

Lung cancer presents with the characteristics of frequent aberrance in driver genes, particularly the epidermal growth factor receptor (EGFR) gene (1). Non-small cell lung cancer (NSCLC) is responsible for ≤80% of all cases of lung cancer, and advanced disease is commonly found at the time of diagnosis. Geﬁtinib and erlotinib are EGFR tyrosine kinase inhibitors (EGFR-TKI) that have exhibited marked therapeutic effects against NSCLC with activating mutations in EGFR, such as exon 19 deletions and L858R point mutations (2). However, resistance will eventually develop in all patients after varying periods of time. Acquired resistance to gefitinib is most commonly conferred upon a patient by the *EGFR* T790M mutation, which has been detected in 50% of NSCLC cases with acquired resistance and in cell line models that have been selected for gefitinib resistance (3). Due to the limited treatment options available for individuals with advanced lung cancer, a requirement exists for the identification of novel therapeutic strategies.

Traditional Chinese medicine is used as a component of complementary and alternative medicine in the treatment of a number of diseases (4). *Garcinia hanburyi*, a plant belonging to the Guttiferae family, is a small tree that is found distributed throughout India, Cambodia and the southern regions of China (5). *Garcinia hanburyi* exudes gamboge resin, which contains gambogic acid (GA) as its main active ingredient; GA has been introduced as an effective anticancer drug (6,7). The potent anticancer activity of GA is mainly dependent on the resulting activation of the impaired apoptosis pathways via downregulation of telomerase in cancer cells (8). Furthermore, GA strongly inhibits angiogenesis via vascular endothelial growth factor suppression (9). The aim of the present study was to investigate whether a combination of gefitinib and GA administration can overcome *EGFR* T790M-mediated resistance in patients with NSCLC.

## Materials and methods

### 

#### Ethics statement

All experiments were approved by the Animal User and Ethical Committees at Shandong University (Jinan, Shandong, China).

#### Compound

Gefitinib was obtained from Sellech Chemicals (Houston, TX, USA). Gambogic acid was purchased from Santa Cruz Biotechnology Inc. (Dallas, TX, USA).

#### Cell line

The NCI-H1975 EGFR T790M mutation human NSCLC cell line was obtained from the American Type Culture Collection (Manassas, VA, USA). The cells were cultured in RPMI 1640 medium supplemented with 10% fetal bovine serum, 100 U/ml penicillin, 100 mg/ml streptomycin and 2 mmol/l L-glutamine (Invitrogen Life Technologies, Carlsbad, CA, USA), and maintained at 37°C in a humidified atmosphere with 5% CO_2_.

#### Efficacy study in vivo

Female, 7–8-week-old, BALB/C nude mice were purchased from Vital River Laboratories (Beijing, China). The mice were maintained in super pathogen-free conditions and housed in barrier facilities using a 12-h light/dark cycle, with food and water *ad libitum*. The mice were subcutaneously injected with 1×107 NCI-H1975 cells suspended in 100 µl of Matrigel (BD Biosciences, Milan, Italy). The tumor volume (TV) was measured and recorded during the treatment period using the following formula: TV = length × width2 / 2. When the tumor volume reached ~150 mm3, the mice were randomly divided into four groups (n=10 for each group) and received normal saline (vehicle group, daily intravenous injection), gefitinib (100 mg/kg, daily oral administration), GA solution (8 mg/kg, daily intravenous injection) or a combination treatment of gefitinib and GA for 28 days. Gefitinib was dissolved in 0.1% Tween 80 prior to use.

#### Western blot analysis

The tumors in each group were collected 4 h after the last treatment with gefitinib, GA or the combination treatment on day 28 of the efficacy study. Western blotting was performed as described previously (10). Proteins (50 µg) were separated by 12% SDS-PAGE and then transferred onto 0.45-µm polyvinylidine fluoride membranes (Bio-Rad Laboratories, Inc., Hercules, CA, USA). The membranes were blocked in phosphate-buffered saline containing 5% non-fat dry milk for 1 h, then incubated at 4°C overnight with the following rabbit anti-human monoclonal primary antibodies: AKT (dilution, 1:1,000; catalog no. 4685), phosphorylated (p)-AKT (Ser473; dilution, 1:1,000; catalog no. 4058), ERK1/2 (dilution, 1:1,000; catalog no. 4695), p-ERK1/2 (Thr202/Tyr204; dilution, 1:1,000; catalog no. 14474), MEK1/2 (dilution, 1:1,000; catalog no. 13033), p-MEK1/2 (dilution, 1:1,000; catalog no. 2338), Bax (dilution, 1:1,000; catalog no. 5023) and Bcl-2 (dilution, 1:1,000; catalog no. 2870) (Cell Signaling Technology, Inc., Danvers, MA, USA). This was followed by incubation with horseradish peroxidase-conjugated anti-rabbit secondary antibody (EMD Millipore, Billerica, MA, USA).

#### Caspase activity assay

Caspase-3, 8 and 9 activity was evaluated by fluorometric detection of apoptosis with caspase-3 (cat. no. KA0740),-8 (cat. no. KA0756) and -9 (cat. no. KA0761) colorimetric protease kits (Abnova, Walnut, CA, USA) according to the manufacturer's instructions. Briefly, the tumor tissues were obtained and lysed in 100µl cell lysis buffer (Cell Signaling Technology, Inc.), and 200 µg protein was incubated with 5 µl 4 mM pNA-conjugated substrate (DEVD-*p*NA for caspase-3, LETD-*p*NA for caspase-8 and LEHD-*p*NA for caspase-9; Abnova) at 37°C for 2 h. The amount of pNA released was measured at 405 nm using a UV-Vis Flour spectrophotometer (CRAIC Technologies, Inc., San Dimas, CA, USA). Next, tissue lysates were centrifuged at 3,000 × g, resuspended in 100 µl apopain lysis buffer and vortexed gently. The suspensions were frozen and thawed 4–5 times using liquid nitrogen and a 37°C water bath, respectively.

#### Statistical analysis

Data are expressed as the mean ± standard deviation. InStat, version 1.14 (GraphPad Software, Inc., San Diego, CA, USA) was used for statistical analysis, and a two-way unweighted mean analysis of variance test was used to determine the statistical significance. P<0.05 was considered to indicate a statistically significant difference.

## Results

### 

#### Gefitinib in combination with GA treatment has a synergistic effect on gefitinib-resistant NCI-H1975 tumor growth in vivo

**Figure 1.**
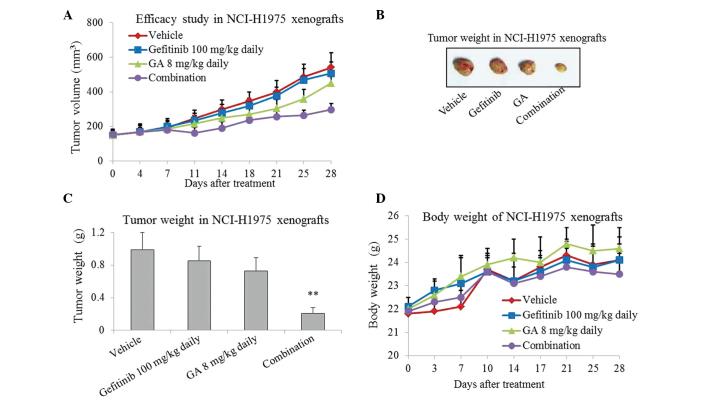
Antitumor activity of gefitinib or/and GA on gefitinib-resistant NCI-H1975 xenografts. Nude mice bearing NCI-H1975 tumors were orally administered 100 mg/kg gefitinib daily or/and an intravenous injection of 8 mg/kg GA daily for up to 28 days. (A) Tumor volume was measured using calipers on the indicated days. (B and C) Tumors weights were measured on day 28. (D) Body weights were measured on the indicated days. Data are presented as the mean ± standard deviation (n=10). **P<0.01 vs. control group. GA, gambogic acid.

In order to investigate the inhibitory effect of gefitinib or/and GA on tumor growth in vivo, gefitinib, GA and gefitinib plus GA were used to treat NCI-H1975 xenografts for 4 weeks. As presented in Fig. 1A–C, treatment with gefitinib or GA for 4 weeks slightly inhibited tumor growth in the NCI-H1975 xenografts. However, more marked inhibition was caused by the combined treatment, which resulted in a ~70% reduction in tumor growth (P=0.008; Fig. 1C). The mice tolerate the single-agent and combination treatments well, with no weight loss or other signs of acute or delayed toxicity (Fig. 1D).

#### Effect of the combined treatment on PI3K and ERK signaling pathways in gefitinib-resistant NCI-H1975 xenografts

**Figure 2.**
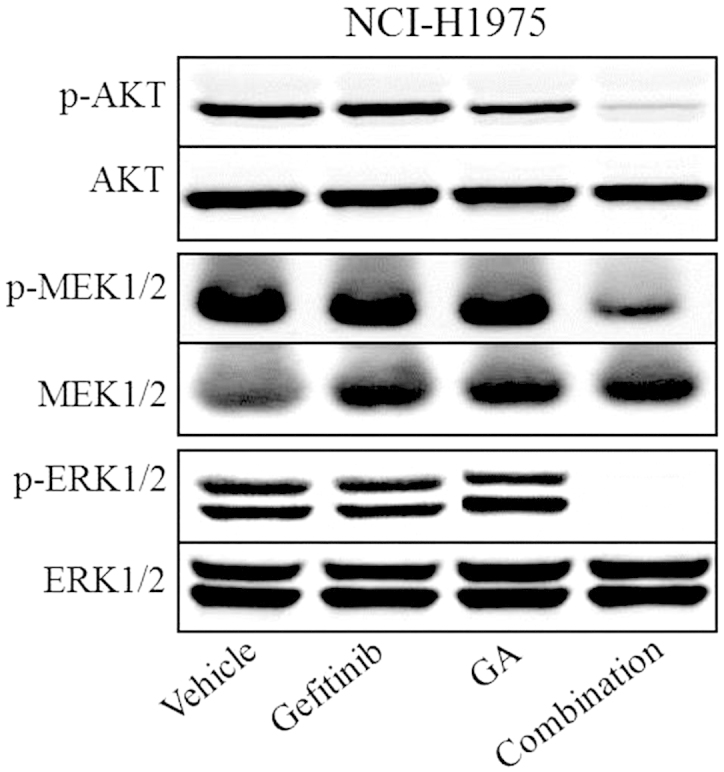
Effects of gefitinib or/and GA therapy on PI3K and ERK pathways. The gefitinib-resistant NCI-H1975 xenografts were treated with gefitinib or/and GA on day 28 of the efficacy study. Tumor tissues were then collected 4 h later to detect levels of phosphorylated (p)-AKT (S473)/AKT, p-ERK (T202/Y204)/ERK (S240/244) and p-MEK/MEK. GA, gambogic acid.

Western blot analysis was used to assess the effect of the two compounds on downstream molecules of the PI3K and ERK pathways. The results showed that p-AKT, p-MEK1/2 and p-ERK1/2 appeared to be inhibited by gefitinib and GA combination treatment, whereas the total protein levels of AKT, MEK1/2 and ERK1/2 remained unchanged in each of the groups (Fig. 2). Western blot analysis also showed that single-agent treatment with gefitinib or GA only exhibited a slightly inhibitory effect on the phosphorylation of AKT, MEK1/2 and ERK1/2 in the NCI-H1975 xenografts.

#### Effect of combined treatment-induced apoptosis in gefitinib-resistant NCI-H1975 xenografts

**Figure 3.**
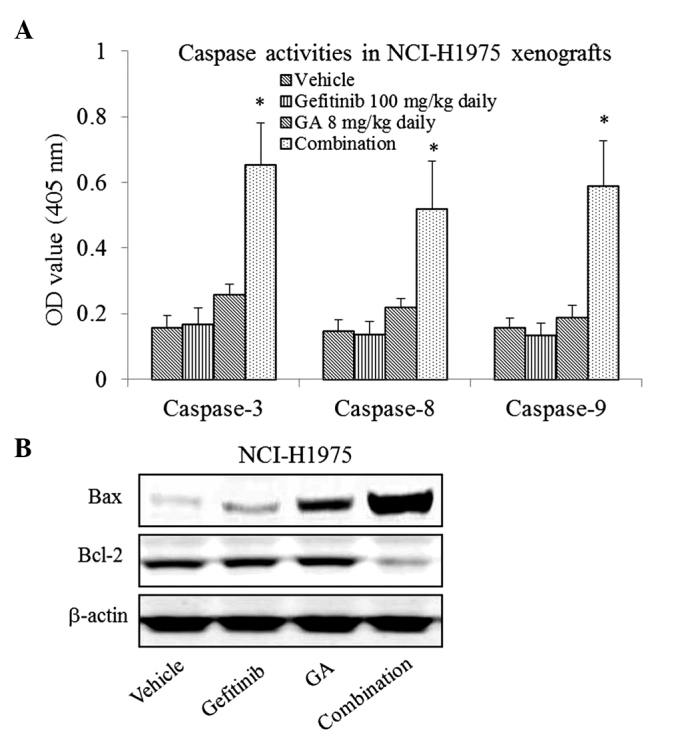
Gefitinib in combination with GA induces apoptosis in gefitinib-resistant NCI-H1975 xenografs. NCI-H1975 xenografts were treated with gefitinib or/and GA on day 28 of the efficacy study. (A) Tumor tissues in each group (n=10) were measured by caspase colorimetric protease kits. *P<0.05 vs. vehicle. (B) The expression of Bax and Bcl-2 was analyzed by western blotting (B). GA, gambogic acid; OD, optical density.

To study whether gefitinib in combination with GA would induce apoptosis in the gefitinib-resistant NCI-H1975 xenografts, the activity of caspase-3, 8 and 9 were measured by colorimetric assay. As presented in Fig. 3A, the results indicated that the gefitinib and GA single-agent treatments exhibited no significant effect on caspase-3, 8 and 9 activity in the NCI-H1975 xenografts (P>0.05 compared with the vehicle), whereas the combined treatment led to a significant increase (P<0.05 compared with the vehicle).

The results of western blotting also showed that the expression of Bax was upregulated, whereas the expression of Bcl-2 was downregulated by the combination treatment (Fig. 3B).

## Discussion

The resistance to the EGFR-TKIs gefitinib and erlotinib ultimately develops in all patients with metastatic EGFR mutant lung cancer. The most commonly observed mechanism for this involves the acquisition of cells harboring a second-site mutation, T790M (11). Irreversible EGFR-TKIs, such as BIBW2992, that can overcome the T790M-mediated resistance to gefitinib have been developed (12), however, clinical trials have failed to show that monotherapy with such irreversible EGFR-TKIs leads to any benefit in those patients with NSCLC refractory to gefitinib (13). Thus, effective therapies for these patients are urgently required. Certain studies have demonstrated the significant anti-proliferative and pro-apoptotic effects of GA on a range of human cancer cell types *in vitro* and *in vivo* (14,15). In the current study, gefitinib in combination with GA was found to have a synergistic inhibitory effect on gefitinib-resistant NCI-H1975 tumor growth, whereas single-agent treatment with gefitinib or GA only exhibited a slight inhibitory effect on tumor growth.

It is established that apoptosis caused by mitochondria is involved in the activation of caspases and Fas-associated death domain protein activation. In the former case, caspase-9 is activated by mitochondrial permeability transitions (ψm), which are mediated by cytochrome *c* release and a reduction in the Bcl-2/Bax ratio (16). In the latter case, Fas-associated death domain protein activates caspase-8, which in turn activates downstream executioners caspase-3 or -7. Xu *et al* (17) reported that GA causes the induction of mitochondria-dependent apoptosis via Bcl-2 and Bax modulation in mantle cell lymphoma JeKo-1 cells. However, in the current study, single-agent treatment with GA could not induce apoptosis in the NCI-H1975 xenografts. It was notable that the combined treatment caused significantly increased levels of caspase 3, 8 and 9 activity, and that an increased expression ratio of Bax/Bcl-2 was also observed in the tumor tissues. The detailed mechanisms behind this require further investigation.

In conclusion, the enhancement of apoptosis caused by treatment with the combination of gefitinib and GA was effective in the suppression of gefitinib-resistant tumor growth caused by a *EGFR* T790M secondary mutation. These findings provide a promising future strategy for the treatment of gefitinib-resistant NSCLC.
